# Epilepsy as a Presentation of a Neuroglial Cyst Associated with Dysgenesis of Corpus Callosum in a Child

**DOI:** 10.1155/2021/6675071

**Published:** 2021-01-21

**Authors:** Outznit Mustapha, Nazik Allali, Chat Latifa, Siham El haddad

**Affiliations:** Pediatric Radiology Department, Rabat Children's Hospital, Avicenne University Hospital, Morocco

## Abstract

Neuroglial (glioependymal) cyst is a rare congenital tumor of the central nervous system usually found in childhood. It can be isolated or associated with other brain malformations. Magnetic resonance imaging is the technique of choice for making the diagnosis. We report the case of a 10-year-old child who presented with epileptic seizures revealing a neuroglial cyst and dysgenesis of the corpus callosum.

## 1. Introduction

The neuroglial cyst, also called the glioependymal cyst, is a benign cystic tumor rarely described in the literature. It is seen mainly in children but also in adults.

The mode of revelation is variable depending on its location in the central nervous system. These lesions are sometimes associated with an abnormality in the development of the corpus callosum.

## 2. Case Report

A 10-year-old child, with no particular history, presented for a year with intermittent chronic headaches without visual disturbances with the notion of three spaced generalized tonic-clonic seizures requiring the prescription of an antiepileptic treatment. The neurological examination was normal.

**Figure 1:**
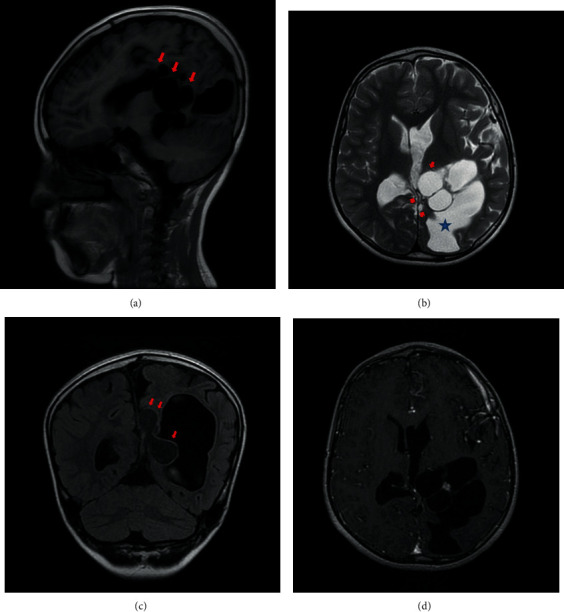
(a) Sagittal slice of T1-weighted image showing the cyst in low signal (arrows). (b) Axial slice of T2-weighted image showing high-signal cyst (arrows) compressing the interventricular foramen responsible for a ventriculomegaly (star). (c) Coronal slice of T2-flair image showing a discreet high signal of the cyst (arrows) compared to the CSF. (d) Axial slice T1-weighted image after gadolinium injection showing the absence of enhancement of the cyst (arrows).

Cerebral magnetic resonance imaging showed an interhemispheric cystic formation, with smooth and well-defined contours, with fluid content in low signal on T1-weighted image signal (Figure 1(a)) and high signal on T2-weighted image (Figure 1(b)) and discrete high signal T2-FLAIR weighted image compared to the cerebrospinal fluid (Figure 1(c)). The cyst was not enhanced after injection of gadolinium (Figure 1(d)).

**Figure 2:**
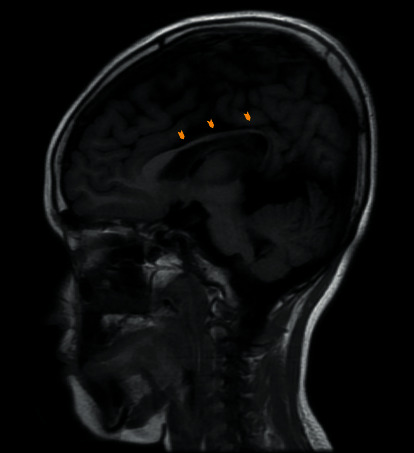
Sagittal slice of T1-weighted image showing dysgenesis of the corpus callosum (arrowheads).

This cystic formation compresses the interventricular foramen responsible for ventriculomegaly of the left lateral ventricle (Figures 1(b), 1(c), and 1(d)). On the sagittal slices, we note the presence of an anomaly in the development of the corpus callosum which is atrophied at the level of its body part (Figure 2).

The cyst was treated by endoscopic fenestration with good clinical outcomes.

## 3. Discussion

Neuroglial cyst is a rare brain tumor also called the glioependymal cyst. It can develop intraparenchymal, in the ventricles, in the subarachnoid space, and rarely in the spinal cord [1]. This type of tumor, developed from embryonic remains, represents less than 1% of all cystic lesions in the brain [2].

Macroscopically, this tumor is surrounded by a thin translucent membrane with a fluid content having a composition very close to that of cerebrospinal fluid (CSF). Its wall is composed of an internal layer made of ependymal cells with secretory activity explaining the progressive growth of the cyst [1].

The clinical symptoms depend on the size and location of the tumor. It can manifest as seizures, motor deficit, chronic headache, psychomotor delay, or macrocephaly in infants [3]. In addition, the neuroglial cyst can be discovered incidentally [3]. It can be isolated or associated with other brain malformations such as agenesis of the corpus callosum, which is the most common abnormality [3, 4].

On imaging, the typical appearance of a neuroglial cyst is that of a large periventricular or interhemispherical lesion with a density and signal identical to CSF on computed tomography and magnetic resonance imaging. In some cases, with the T2 sequence, the fluid content may have a higher signal than that of the CSF (indicating a fluid rich in proteins). The wall of the cyst does not enhance after injection of gadolinium [4].

The diagnosis can be made during the prenatal period by morphological ultrasound [3] [4], allowing close monitoring of these patients and early management after birth to avoid neurological sequelae.

Histopathological analysis of the tumor is not necessary to make the diagnosis because the MRI radiological semiology of the lesion and its association with the abnormalities of the corpus callosum are sufficient to make this diagnosis [5].

The differential diagnoses of neuroglial cysts are arachnoid cyst, epidermoid cyst, ependymal cyst, and porencephalic cyst [5]:

- Arachnoid cyst typically presents as an unilocular extra-axial cystic lesion which has the same signal as CSF, its outlines are sharp and smooth, and it can displace or deform adjacent brain or adjacent calvarium (scalloping). The arachnoid cyst does not enhance [6]
- Epidermoid cysts appear as lobulated CFS-like mass that fills and expand CSF spaces and exerts a gradual mass effect, insinuating between structures and encasing adjacent nerves and vessels. Calcification is present in 10%–25% of cases, and a thin enhancement around the periphery may sometimes be seen [6].
- Ependymal cysts are rare benign neuroepithelial cysts lined by ependymal cells, typically nonenhancing periventricular lesion [6]
- Porencephalic cyst is a rare condition of CSF accumulation within the brain parenchyma that communicates directly with the ventricular system. It appears as a CFS-like lesion lined by white matter, without mass effect on the adjacent parenchyma or enhancement with contrast [7] [8]

For symptomatic patients, treatment is surgical. There are many methods used: a cysto-peritoneal or cysto-ventricular bypass, partial or total resection [2]. Neuroendoscopic cyst fenestration is a minimally invasive technique of low morbidity with very short hospitalization and long-term efficacy [5]. For small, asymptomatic cysts discovered incidentally, surgical treatment is not necessary and monitoring is recommended [5].
